# LSTM-Based Path Prediction for Effective Sensor Filtering in Sensor Registry System

**DOI:** 10.3390/s21238106

**Published:** 2021-12-03

**Authors:** Haotian Chen, Sukhoon Lee, Byung-Won On, Dongwon Jeong

**Affiliations:** 1 Department of Software Convergence Engineering, Kunsan National University, Gunsan 54150, Korea; chenhaotian@hgu.edu.cn (H.C.); leha82@kunsan.ac.kr (S.L.); bwon@kunsan.ac.kr (B.-W.O.); 2 College of Information and Engineering, Hebei GEO University, Shijiazhuang 050031, China

**Keywords:** path prediction, LSTM, Monte Carlo, sensor registry system

## Abstract

The Internet of Things (IoT) is expected to provide intelligent services by receiving heterogeneous data from ambient sensors. A mobile device employs a sensor registry system (SRS) to present metadata from ambient sensors, then connects directly for meaningful data. The SRS should provide metadata for sensors that may be successfully connected. This process is location-based and is also known as sensor filtering. In reality, GPS sometimes shows the wrong position and thus leads to a failed connection. We propose a dual collaboration strategy that simultaneously collects GPS readings and predictions from historical trajectories to improve the probability of successful requests between mobile devices and ambient sensors. We also update the evaluation approach of sensor filtering in SRS by introducing a Monte Carlo-based simulation flow to measure the service provision rate. The empirical study shows that the LSTM-based path prediction can compensate for the loss of location abnormalities and is an effective sensor filtering model.

## 1. Introduction

The Internet of Things (IoT) is a crucial component of the new generation of information technology. As its name indicates, IoT is a network that extends on the Internet, with terminals that expand to objects for data exchange and communication. It is designed for seamless communication and data transfer between interrelated heterogeneous devices [1]. The sensor network, for emphasis on wireless, also known as wireless sensor networks, is an essential branch of IoT research. It utilizes sensors to collect dynamic environmental data, delivers observations to edge computing devices for analysis and evaluation, and finally provides intelligent services. In recent years, with the rapid spread of inexpensive sensors and wireless equipment, more and more services based on sensor networks have been proposed, such as health care [2], natural calamity monitoring [3], and intelligent agriculture [4].

In a sensor network, sensors collect heterogeneous data. To combine various data, the edge node, such as the mobile device, must be aware of the metadata that defines the properties of the data. There are many ways to accomplish this, such as defining data attributes directly in the framework [5], expressing data properties by ontology [6], and so on. A sensor registry system (SRS) [7] based on ISO/IEC11179, is a suitable solution. It provides semantic interoperability between sensors and devices for a ubiquitous computing environment. The data acquisition process with SRS consists of two connections. The first connection is between the mobile device and SRS. The mobile device fetches the metadata of the sensor through the first connection. Based on intelligent service requirements and the received metadata of ambient sensors, the mobile device sends requests to the corresponding sensors and collects the sensor data. This is the second connection.

Existing optimization methods for sensor filtering in SRS [8,9,10] focus on the first connection, i.e., how to improve the reliability of acquiring metadata. There is a denial-of-service area in which the mobile device cannot communicate with SRS in these efforts. When a user enters a denial-of-service area, the mobile device cannot establish the first connection in the previous text and is unable to fetch the metadata of the ambient sensors. Such a situation may result in the inability to provide intelligent services. The movement pattern of humans is predictable [11]. Therefore, the authors introduce the path prediction algorithms that predict the next position of the user. Suppose the user is going to enter a denial-of-service area. In that case, the metadata is obtained before entering the area, and the service provision rate [9] of SRS can be improved.

However, there are disadvantages to these efforts. 1. The denial-of-service area is grouped by measuring the reference signal received power (RSRP) of cellular networks within the research area [9,10]. In recent years, with the popularity of commercial 4G services and the improvement of cellular network infrastructure, the quality of service between SRS and mobile devices can be guaranteed. There is no denial-of-service area in daily life. The sensor filtering module that handles the denial-of-service area is no longer necessary. 2. These works do not quantitatively evaluate the performance of sensor networks with SRS. At that time, there was no historical trajectory dataset for SRS. The service provision rate, which indicates the performance of SRS, was directly calculated by the algebraic expressions [8]. 3. The mobile device’s position obtained from GPS is sometimes wrong. GPS drift is the difference between the actual position and the position recorded by a GPS receiver. Consumer-grade GPS receivers (such as mobile devices) are not 100% accurate, which usually causes a difference between the actual and recorded positions. The traditional sensor filtering module in SRS assumes that the input position is actual and cannot handle GPS drift. Suppose the GPS of the mobile device receives a wrong position, it may cause SRS to send the wrong sensor metadata to the mobile device, and thus, service failure occurs.

To address these concerns, we propose a dual collaboration strategy for effective sensor filtering in SRS and quantitatively evaluate the performance of SRS by a Monte Carlo-based service provision rate simulation flow. There are two main advantages over existing works. First, we focus on sensor filtering with GPS drift. A dual collaboration strategy is proposed. It employs an LSTM-based path prediction algorithm to predict the current position from the historical trajectory. Both positions predicted by the historical trajectory and read from GPS are used to improve the performance of sensor filtering in SRS. The metadata of the sensors at both positions is sent to the user’s mobile device. If one of the positions is correct, the mobile device can connect to the actual ambient sensors and acquire heterogeneous data. Second, we design a service provision rate simulation flow based on the Monte Carlo method [12]. In the past two years, we collected movement data from 59 participants [13]. It recorded a wide range of users’ daily activities. The simulation flow randomly generates sensors and uses the collected historical movement to model the service provision rate, which is an ideal method for evaluating the performance of sensor filtering. The results show that a dual collaboration strategy that simultaneously collects GPS readings and predictions from historical trajectories can effectively improve SRS performance. In particular, the LSTM-based model has the highest accuracy in path prediction and can bring the service provision rate approaching the theoretical performance of SRS.

The remainder of the paper is organized as follows. Section 2 gives an overview of the existing IoT-based sensor systems and path prediction algorithms. Section 3 describes the LSTM-based path prediction algorithm, the service provision rate simulation flow, and the dual collaboration strategy. Section 4 evaluates the geo-embedding, the LSTM-based path prediction, and the service provision rate in SRS. Section 5 concludes the paper.

## 2. Related Work

Research on IoT-based sensor systems has a long history. Since sensors can detect heterogeneous data, such multi-modal sensor systems are generally used in environmental monitoring. The environment may be harsh, such as in nuclear waste containers [14]. It also may be large, such as sensing the energy consumption of air conditioners in several buildings [5] or landslide monitoring on a hillside [15]. In these works, the metadata of sensors is globally pre-defined. The acquired data is transferred to the IoT platform through the wireless network for further visualization and data mining services. However, in large-scale IoT networks, to relieve the load on the IoT platform, a portion of storage and computation tasks are allocated to the edge devices [16]. In Gimpo-si, an IoT-based sensor service was built for crime prevention [17]. The edge computing device, such as a mobile device, cannot afford to store the metadata of all the sensors in a medium-sized city. The metadata of the sensors is locally visible. Therefore, a location-based SRS is used for the interoperability of the heterogeneous sensor data in large-scale IoT-based sensor systems [9].

In this work, we improve the effectiveness of sensor filtering in SRS by using a dual collaboration strategy. On the one hand, the strategy provides metadata of ambient sensors by acquiring the position of the mobile device in real-time. On the other hand, it compensates for the losses caused by GPS errors through a path prediction algorithm with historical trajectories. As the name implies, the path prediction algorithms predict the future position of an object. A high accuracy path prediction algorithm is vital to compensate for GPS errors and improve the performance of the sensor filter in SRS.

Manually extracting frequent patterns is the most basic way of handling such problems. Giannotti et al. [18] define frequency pattern as using a similar time to pass through the same sequences of locations to construct visiting density in both temporal and spatial domains. He et al. [19] restructure the route data based on time of the day to separate time property and repeatedly compare the regular coefficient with the threshold to group frequency-based regular routes.

A more convenient approach is to employ a tree structure to represent frequency-based behavior patterns [20]. Chen [21] et al. present continuous route pattern mining. They first divide the research area into cells and map the GPS data into cell-temporal sequences. Then, referring to the idea of frequency-based clustering with thresholds, the cell-temporal sequence composed of cells is transformed into a regional-temporal sequence formed by regions-of-interest. Finally, the regional-temporal sequence is used to organize a tree structure to predict the result. This method reduces the number of nodes through projections. It can also reuse data to implement an incremental mining strategy to reduce the computational load on the mobile device. Tiwari et al. [22] further optimize the prediction speed by parallelizing the tree prediction problem through a partial match.

The basic probabilistic model is the Markov model, it assumes that the future state of a given temporal-spatial sequence depends only on the current state. In path prediction, its principal problem is how to define the state manually with multi-modal data, such as position [23,24], semantics, and context [25]. Lee et al. [8] equate the position to path fragment with pre-defined roads. The state is the fragment that contains location information. The transition probability matrix is determined by the frequency of moving from one fragment to another. Gambs et al. [26] integrate historical data into *n* previous visited points of interest. Each point of interest corresponds to a state in the Markov model. In addition to integrating *n*-step data into a single state, the sequence can also be processed using the higher-order Markov model [27] or Hidden Markov Model (HMM). However, for HMM, the Baum–Welch approach is not effective [28] and still requires the manual design of the hidden state [29,30].

Recurrent neural networks (RNN) are effective tools for processing sequential data and can be applied to path prediction as well [31,32]. Crivellari et al. propose a series of methods to analyze call detail records related to tourists’ behavior in Italy, such as geo-embedding [33,34], predicting individual mobility traces [35], trajectory translation [36], and urban traffic forecasting [37]. Similar to natural language processing, these methods have three significant components [38]: 1. Converting the original data into high-dimensional dense vectors by word2vec [39,40]; 2. Feeding the data into an LSTM encoder; 3. Predicting the results with a decoder depending on the application [41].

Nevertheless, Markov models struggle to extract higher-order or irregular transition patterns. RNN cannot detect periodicity in sequences. The attention mechanism [42] can also be used in path prediction. Feng et al. [43,44] propose DeepMove contained in multi-modal embedding for a fusion of various factors, a historical attention model to capture multi-level periodicity, and a decoder for multi-task prediction. H-TALL [45] concerns the linkage between destinations through an attention mechanism and utilizes multiple prediction layers to improve the accuracy by combining different granularity. Altaf et al. [46] focus on temporal difference and positional discrepancy using two independent attention units, respectively. The results show that the reasonable value of the context window occupies an essential role in hyperparameter tuning of the attention mechanism.

## 3. Methodology

### 3.1. Problem Definition

This article aims to improve sensor filtering in SRS [7]. SRS is a framework of sensor networks for semantically interpreting and processing heterogeneous information. The concept of SRS is inherited from ISO/IEC 11179 – Metadata Registry, which is one of the international standards used to develop the sharing and exchange of data. Based on the combined use of metadata registry and sensor network, users can directly handle the heterogeneous sensor data through mobile devices in SRS.

There are two connections in SRS. One is the mobile network for a mobile device to receive registered sensor metadata. The other is the sensor network for the mobile device to connect directly and obtain data from sensors. In the applied scenario [10], the research area Ω is divided into grid cells c. $L_{c}$ is a list of sensors *s* linked to the cell *c*. The trajectory of human movement is a sequence composed of a series of cells $\left\{c_{1},\cdots ,c_{t-1},c_{t}\right\}$, where the superscript indicates the time, $c_{t}$ is the current cell of the user, and $c_{1}$ is the starting cell of the trajectory. When a mobile device lies inside the cell $c_{t}$, it gets the metadata of sensors in the list $L_{c_{t}}$ via the mobile network. It is the first connection in SRS. After the list of ambient sensors is obtained, the mobile device connects to sensors according to the metadata. It is the second connection.

Existing sensor filters in SRS mainly focus on the unstable connection of the mobile network. There is an area without mobile network coverage called the denial-of-service area. When a user moves into a denial-of-service area, the mobile device fails to obtain the metadata of sensors. A path prediction algorithm predicts the user’s next position $c_{t+1}$. By the strategy of obtaining the metadata of sensors in the future cell, users can handle data from ambient sensors in the denial-of-service area.

This work focuses on the second connection, which connects the mobile device to the sensor. GPS sometimes shows users in the wrong place. Many things can degrade GPS positioning accuracy. Common causes include satellite signal blockage due to buildings, indoor or underground use, and signals reflected off buildings or walls. In SRS, users read their position at regular intervals and then connect to ambient sensors. There are many ways for mobile devices to connect to ambient sensors, such as Wi-Fi, Bluetooth, ZigBee. However, if the current position from GPS is wrong, the mobile device cannot access the actual sensors. It is a failed sensor filter. To address this issue, we employ a dual collaboration strategy to improve sensor filtering in SRS. Both positions predicted by the historical trajectory $\left\{c_{1},c_{2},\cdots ,c_{t-1}\right\}$ and read from GPS values are used to guess the user’s current cell $c_{t}$. In this way, blind spots caused by GPS errors can be filled.

### 3.2. Trajectory Preprocessing

The data collected by the GPS device is a collection of spatial-temporal points. Each spatial-temporal point *P* is a tuple of geographical coordinate *g* and timestamp *t*, i.e., $P=(g_{lon},g_{lat},t)$. Due to the uncertainty of the data obtained from GPS devices, outliers need to be removed. We use a sequential model that consists of a range filter, time filter, long-distance filter, speed filter, short distance filter, sequenced DBSCAN, and angle filter to refine the data received from GPS devices [13]. We then organize the smooth data as a set of trajectory sequences in the form of $\left\{S_{i}|i=1,2,3,\cdots ,k\right\}$. Trajectory sequence *S* is a spatial-temporal point sequence expressed as $S=\left\{P_{i}|i=1,2,3,\cdots ,n\right\}$, where *n* is the number of spatial-temporal points.

**Figure 1:**
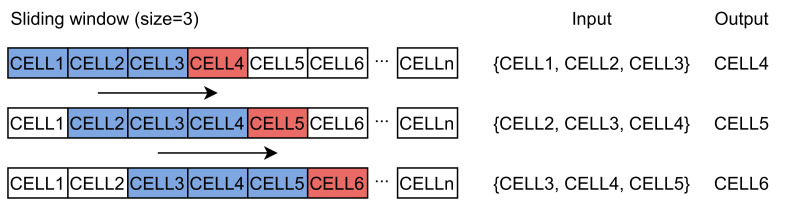
The process of the sliding window (with a length of three cells in the past) and the model input–output pairs.

In this work, we use a regular grid to divide the research area into cells. Grid-based path prediction is more flexible than the segment-based method because the learned and predicted results are not limited by the road network structure. Spatial-temporal points are mapped to cells with a non-injective surjective system. After the mapping process, trajectory sequences are transformed into discrete sequences with cells, expressed as $S^{\prime }=\left\{c_{i}|i=1,2,3,\cdots ,n\right\}$. We use these trajectory sequences to train the word2vec model to obtain dense vectors representing the behavior pattern of cells. However, the sequences cannot be directly put into the LSTM model. Each sequence should be divided into trajectories *T* with fixed length expressed as $T=\left\{c_{i}|i=1,2,3,\cdots ,m\right\}$, where *m* is the size of the sliding window. The window size depends on the final purpose. A larger window increases the influence of long-term historical movement trajectory on the predicted results. Conversely, a smaller window size reinforces the effect of the last cell. The process is represented in Figure 1. After the sliding window, we can obtain the input data and labels used for training the neural network.

### 3.3. Word2vec Model

Embedding is an injective, surjective structure to use a dense vector for representing an object. Its primary function is to convert sparse vectors, such as one-hot encoding into dense vectors, which are convenient for the processing of the deep neural network. The embedding vector can represent features of the corresponding object, and the distance (cosine similarity) between the vectors reflects the similarity between the objects. Compared with the feature vector generated by traditional methods such as Matrix Factorization, embedding is more expressive. The popularity of this concept began with the study of using a vector to represent a word in training corpora.

Word2vec [39,40] is a popular model that generates vector expressions for words. Since it was proposed in 2013, embedding technology has been extended from natural language processing to other fields, such as advertising, searches, images, and recommendation systems. It has become a crucial technical point in the deep learning knowledge framework. Applied to the field of geographic information mining, it is also known as geo-embedding [33].

**Figure 2:**
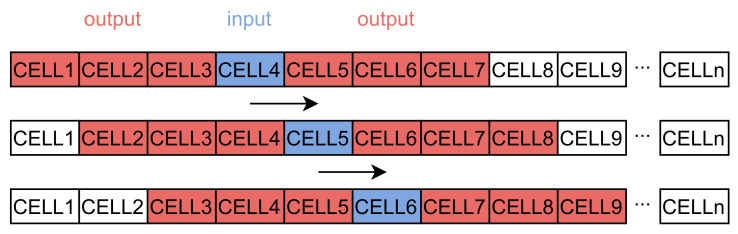
Graphic representation of the skip-gram model.

We use a corpus consisting of a set of trajectory sequences to train the word2vec model. Each cell in the research area is initialized with a random vector of a pre-defined size to obtain the initial embedding matrix of dimensionally num_size×vector_size. Specifically, we adopted the skip-gram approach. Suppose a trajectory sequence of length *n* is $S^{\prime }=\left\{c_{i}|i=1,2,3,\cdots ,n\right\}$, each cell in the sequence determines its neighboring cells. A graphic representation of skip-gram is in Figure 2. The skip-gram approach utilizes a sliding window to acquire training instances. For each trajectory sequence in the corpus, a fixed-length training instance is obtained by sliding through a window of length 2w+1. The input is the current cell vector. Since each cell determines the neighbors, based on the maximum likelihood estimation method, the product of conditional probabilities of all samples is desired to be maximized. The cost function is the negative log probability of the current answer and is defined over the entire dataset. After several iterations of training the whole corpus and updating the embedding vector values, the dense vectors used to represent cells are obtained.

### 3.4. LSTM for Path Prediction

RNN is a sort of neural network for processing sequential data. Compared with general neural networks, it can handle data with sequential changes. LSTM [47] is a variant of RNN. It is designed to solve the gradient vanishing problem to perform practical computation without long-range decay and controls the update of the cell state *c* and hidden state *h* using the gate mechanism. Equations (1)–(6) report the updating formulations.

(1) $$\begin{array}{cc} i_{t} & =\sigma \left(W_{ix}x_{t}+W_{ih}h_{t-1}+b_{i}\right) \end{array}$$

(2) $$\begin{array}{cc} f_{t} & =\sigma \left(W_{fx}x_{t}+W_{fh}h_{t-1}+b_{f}\right) \end{array}$$

(3) $$\begin{array}{cc} o_{t} & =\sigma \left(W_{ox}x_{t}+W_{oh}h_{t-1}+b_{o}\right) \end{array}$$

(4) $$\begin{array}{cc} g_{t} & =\tanh\left(W_{gx}x_{t}+W_{gh}h_{t-1}+b_{g}\right) \end{array}$$

(5) $$\begin{array}{cc} c_{t} & =f_{t}*c_{t-1}+i_{t}*g_{t} \end{array}$$

(6) $$\begin{array}{cc} h_{t} & =o_{t}*\tanh\left(c_{t}\right) \end{array}$$

Equation (1) is the input gate. The current input $x_{t}$ has the weight $W_{ix}$, the previous hidden state $h_{t-1}$ has the weight $W_{ih}$, and $b_{i}$ is the bias. The input gate computes a weighted sum of the current input and previous state, passes that through a sigmoid function, and gets the output $i_{t}$. $i_{t}$ is the mask to select the information to input to the current cell state $c_{t}$. Similarly, Equations (2) and (3) use the same current input $x_{t}$ and the previous state $h_{t-1}$ with corresponding weights $W_{fx}$, $W_{fh}$, $W_{ox}$, and $W_{oh}$ and biases $b_{f}$ and $b_{o}$ to obtain the masks $f_{t}$ and $o_{t}$. $f_{t}$ is the output of the forget gate (2). This gate aims to delete information from the current cell state $c_{t}$ when it is no longer needed. $o_{t}$ from the output gate (3) is used to decide what information is required for the current hidden state $h_{t}$. *g* indicates the new information to be added to the current cell. It is also extracted from the current input $x_{t}$ and the previous hidden state $h_{t}$ with weights $W_{gx}$ and $W_{gh}$ and bias $b_{g}$. Equation (4) extracts the substantive information usually using tanh as the activation function. ∗ is the Hadamard product. In Equation (5), the previous cell state $c_{t-1}$ is filtered by the forget gate output $f_{t}$ and the input mask $i_{t}$. The new hidden state $h_{t}$ is a filtered version of the new cell state $c_{t}$, resulting from the multiplication between the output mask $o_{t}$ and the tanh of the cell state $c_{t}$. In RNN, the time dependency is directly stored in the hidden state and passed to the next step. In LSTM, the data is further gated to decide whether to enter the cell state or not. In short, the forget gate $f_{t}$ determines if the time dependency from the previous cell state is retained in the current cell. The data filtered by the input gate and forget gate are stored in the cell state. It is conditioned by the output gate to acquire the current hidden state.

**Figure 3:**
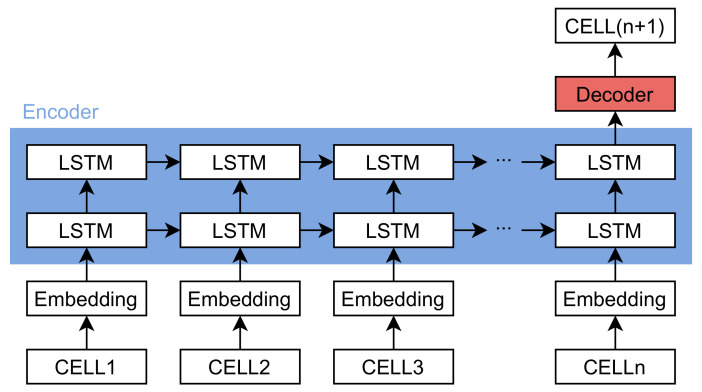
Overview of the neural network model with LSTM for next cell prediction.

In this work, we utilize LSTM to predict the next position from historical trajectories. Its essence is a multi-classification problem on sequential data. The model consists of three components: an embedding layer, an LSTM layer, and a softmax decoder. Each cell identifier is first converted through the embedding layer into a dense vector representing the behavioral characteristics. After embedding, the fixed-length trajectories are then fed into the LSTM for learning the long-range dependencies of the sequences. After extracting the information by the LSTM encoder, the next step is to predict the result. In the softmax decoder, similar to the decoder of the general multi-classification problem, we stack the dropout layer, fully connected layer, and softmax layer to estimate the probability of the next cell. A graphic overview of the model is illustrated in Figure 3.

### 3.5. Service Provision Rate Simulation Flow

Service provision rate $R_{SP}$ is the probability of successfully providing services to a mobile device when they are requested. As mentioned earlier, we divide the research area into 10×10 m cells. The position of a mobile device is mapped to the corresponding cell and sent to SRS in real-time. A dual collaboration strategy based on LSTM-based path prediction is employed to improve the performance of sensor filtering. The performance refers to the probability of successful requests between mobile devices and sensors. When a user’s position is confirmed, SRS provides metadata of potentially connectable ambient sensors. The user’s mobile device attempts to connect to these sensors for data, which is a service request. If the mobile device can successfully connect to the corresponding sensor, SRS has provided a service. Conversely, if for some reason the link cannot be established, it is a failed service. A function can be defined to describe the state of the service request.

(7) $$\begin{array}{c} \Phi \left(c,s\right)=\left\{\begin{array}{c} 1,\mathrm{SRS}\;\mathrm{provides}\;a\;\mathrm{service}\;\mathrm{when}\;\mathrm{the}\;\mathrm{user}\;\mathrm{is}\;\mathrm{in}\;\mathrm{cell}\;c.\;\\ 0,\mathrm{it}\;\mathrm{is}\;a\;\mathrm{failed}\;\mathrm{service}\;\mathrm{when}\;\mathrm{the}\;\mathrm{user}\;\mathrm{is}\;\mathrm{in}\;\mathrm{cell}\;c.\; \end{array}\right. \end{array}$$

where *c* is a cell in the research area, and *s* is a potentially connectable ambient sensor of the cell *c*. As detailed later, the function Φ(c) is implemented through a probabilistic communication model. In some cases, a cell has no corresponding sensor, which means that the user does not need SRS service in this cell. Such a cell will be skipped in the service provision rate calculation. On the contrary, a cell may be associated with multiple sensors. We use the average of the states for numerous sensors to describe the count of service successes at the cell. The count *r* is not necessarily an integer but may also be a float number. Suppose cell *c* corresponds to *m* sensors. Its count of service successes is as follows.

(8) $$\begin{array}{c} r_{c}=\frac{1}{m}\sum_{i=1}^{m}\Phi \left(c,s_{l}\right) \end{array}$$

The service provision rate $R_{SP}$ is the percentage of provided services within a trajectory $\left\{c_{i}|i=1,2,3,\cdots ,n\right\}$, which is the cell version for network availability.

(9) $$\begin{array}{c} R_{SP}=\frac{1}{n}\sum_{i=1}^{n}r_{c_{i}} \end{array}$$

To quantitatively evaluate the effectiveness of the dual collaboration strategy in sensor filtering, we have developed a Monte Carlo-based simulation flow in which $R_{SP}$ is a metric for evaluation. The basic idea of the Monte Carlo method is: first, build an appropriate probability distribution so that the solution to be obtained is equal to the mathematical expectation value of the random variable; second, conduct a simulation experiment and repeat it several times to derive the random event; finally, statistically average the results of the random investigation to obtain the solution of the target. The proposed service provision rate simulation flow is a two-step approach:

**Step 1**. Generate random sensors.

**Step 2**. Evaluate the service provision rate with historical trajectories.

**Figure 4:**
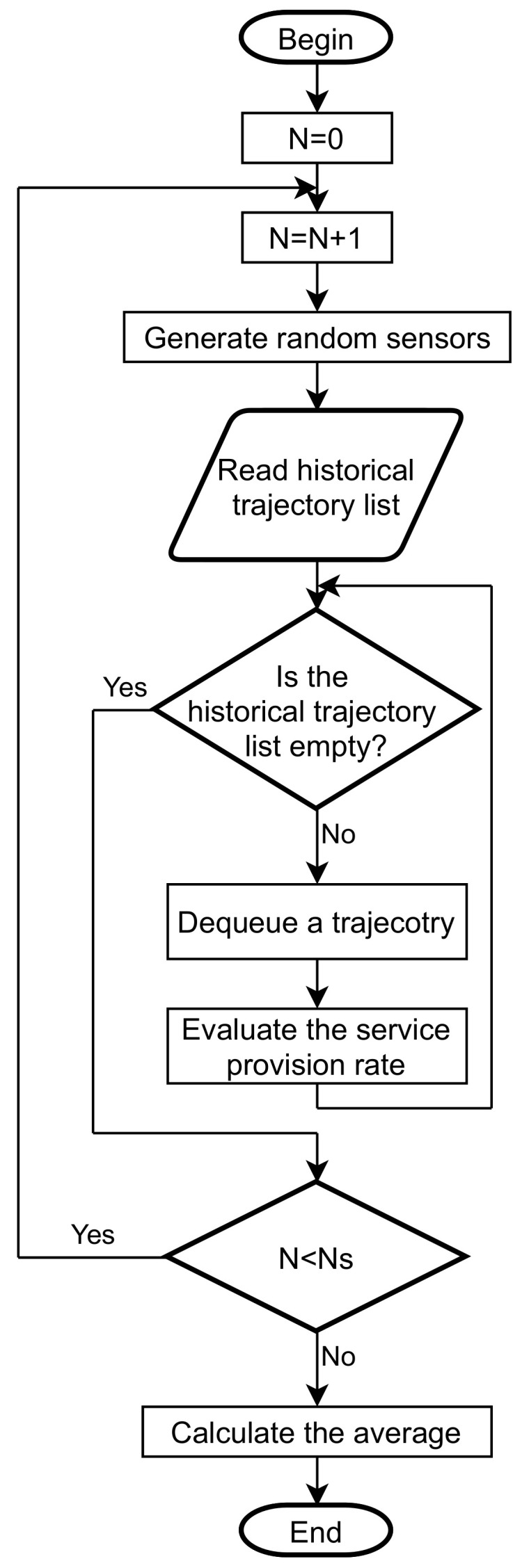
Service provision rate simulation flow.

All the above steps are repeated several times, as shown in Figure 4. $N_{s}$ is the total number of simulation runs. After $N_{s}$ rounds, we calculate the average of the results of each round as the final service provision rate.

**Figure 5:**
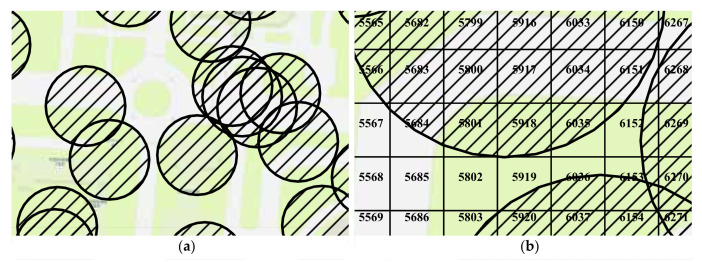
Examples of the communication disks. (**a**) The communication disks are represented as circles with hatch fills. (**b**) The intersection areas of the cells and communication disks are different. The area ratio is defined as the intersection area in the cell divided by the area of the cell. It implies that we can identify the connectivity by the area ratio.

Sensing and communication are two essential concepts in the study of sensor networks [48]. For a sensor *s*, its sensing area is a disk, denoted by $disk_{R_{s}}\left(s\right)$. Its boundary is called the sensing circle, denoted by $circle_{R_{s}}\left(o\right)$, where $R_{s}$ is the sensing radius. Similar to sensing, each sensor has a communication area, which is also a disk. Within the communication radius $R_{c}$, its communication disk is $disk_{R_{c}}\left(s\right)$, and the boundary of the communication disk of sensor *o* is $circle_{R_{c}}\left(o\right)$. In this work, we assume that the sensors already cover the target area as required and focus on the connectivity between a mobile device and the sensors. We generate two random numbers using a uniform distribution on the interval (0,1). These two random numbers are multiplied by a factor to create the longitude and latitude of the origin of the sensor, respectively. In step 1, $n_{s}$ sensors are generated randomly with communication radius $R_{c}$, as shown in Figure 5a. The $n_{s}$ and $R_{c}$ are hyperparameters that can be set according to requirements. The PostGIS is employed for spatial operations, which is a spatial database extender for PostgreSQL object-relational database. It adds support for geographic objects allowing location queries to be run in SQL. For example, in step 1, the communication boundary for each sensor is a circle resulting from origin and radius. As a spatial database, PostGIS supports operations on spatial objects, such as finding intersections. It implies that we can express the probability of a successful connection by the area ratio of the intersection to the cell.

Figure 5b illustrates the concept of indicating the connectivity by area ratio. For CELL 5800, its entire region is covered by the communication disk, which means that the probability of a mobile device connecting to the sensor is 100%. In contrast, for the CELL5685, its geometry does not intersect any communication disk. The area ratio is 0, and the mobile device will fail to connect to the sensor within the CELL5685. Between these two extremes, it is possible to evaluate whether the service requests from mobile devices in the cell are provided or not by comparing random numbers and area ratios. Let *p* be a random number using a uniform distribution in the interval (0,1), *R* is the area ratio of the intersection to the cell. If p<R, the service request is successful. If not, the service request is failed.

Algorithm 1 describes the evaluation of a service request in the ideal scenario. The ideal scenario means that the GPS is entirely reliable. A mobile device sends the service requests and obtains responses based on its current position in SRS. First, a trajectory is extracted from the historical data for evaluating the service provision rate. The last cell in the trajectory is the current position of the mobile device. Second, the SRS server sends metadata about the ambient sensors to the mobile device. Once the mobile device has received the metadata, it requests a service to each sensor to create a peer-to-peer connection with Wi-Fi Direct and exchange the sensing data. If the connection is established, the service request is successful, and the service success count is increased by 1. If not, the count is not increased. For the cell that intersects with multiple communication disks, we evaluate whether the service request for each sensor is successful or no, and take the average of the success requests as the final count of service successes, as shown in Equation (9).
**Algorithm 1:** evaluate_service_request_ideal_scenario
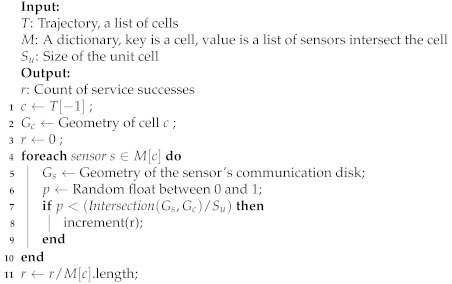


In a real environment, there is a possibility that the position received by GPS will drift into error. The wrong position will reduce the service provision rate of SRS. We use Algorithm 2 to examine this reduction quantitatively. As compared to Algorithm 1, Algorithm 2 has a new hyperparameter GPS reliability $R_{G}$ to denote the probability of mobile devices getting the correct position. Similar to the method used to evaluate whether the network is connected, we employ a random number with a uniform distribution in the interval (0,1) compared to the GPS reliability to assess whether the position provided by the mobile device is accurate at the moment.
**Algorithm 2:** evaluate_service_request_with_GPS_drift
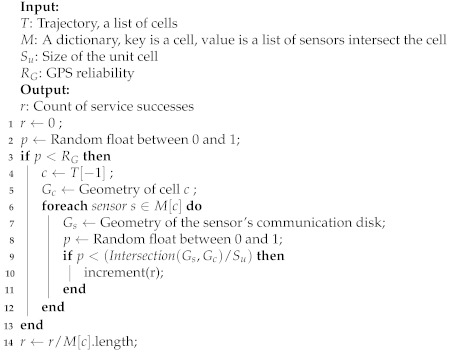


To evaluate the impact of path prediction on service provision rate, we incorporate the path prediction algorithm as a dual collaboration strategy in SRS, as shown in Algorithm 3. The cells obtained from the mobile device and path prediction are processed together. The concatenation of the list of sensors corresponding to the two cells is taken as the service request target. The service provision rate will be achieved after the random simulation.
**Algorithm 3:** evaluate_service_request_with_path_prediction
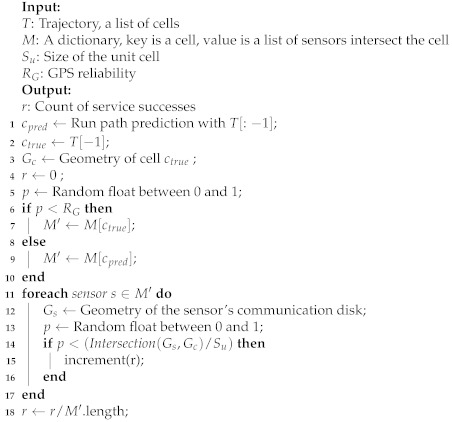


Figure 4 indicates the service provision rate simulation flow. It consists of two nested loops. The inner loop extracts each trajectory in the historical trajectory list. In this way, the SRS performance can be measured under different behavioral patterns. The outer loop iterates over various sensor networks. Sensors are repositioned randomly in the research area. Thus, it is possible to evaluate the performance of SRS under different sensor networks. After achieving the number of simulation runs $N_{s}$, the simulation flow is terminated. We can average the results generated by each step to arrive at the service provision rate of SRS.

## 4. Experiment

### 4.1. Dataset

The ubiquitous smart devices with GPS make the collection of personal trajectories more efficient and effective. An Android application written in Java runs on smartwatches records the spatial-temporal points of the participants. It uses the fused location provider in Google Play services location APIs to receive the device’s last known location every 5 s. Participants wear their smartwatches the whole day, run the application under the open sky, and stop it indoors to save battery power. The entire GPS trajectory dataset collected by 59 users in six months contains over 900 million spatial-temporal points. Because of the requirements for behavior pattern research, the data should be concentrated in a fixed research area. Our research area is the campus of Kunsan National University and living areas near it. After removing the unwanted data, a data preprocessing strategy contains five filters, and the sequenced DBSCAN [13] is employed to remove outliers. The filtered information consisted of 240 million spatial-temporal points that make up 35,234 trajectories with a total distance of 1588 km and 3300+ h. It recorded a broad range of user’s daily activities, such as going to class, shopping, and dining. This fact makes the dataset ideal to validate path prediction algorithms and service provision rate in a sensor register system.

### 4.2. Experimental Settings

We implement the word2vec algorithm by relevant Python modules in the Gensim library, train the skip-gram model with a window size of nine cells in the past and another nine cells in the future. The cells with a total frequency lower than 50 are ignored to deal with many potentially inaccessible and irrelevant places. To measure the “closeness” between embeddings, we use the cosine similarity. Cosine similarity measures the angle between two vectors. The smaller the angle, the larger the cosine similarity, and the more similar the two vectors of cells. As shown in Equation (10), cosine similarity consists of the dot product of unit-normalized vectors.

(10) $$\mathrm{sim}\left(i,j\right)=\cos\left(i,j\right)=\frac{i\;\cdot \;j}{∥i∥\;\cdot \;∥j∥}$$

We design the neural network model using an input embedding size of 100 dimensions and a block of two LSTM layers having a hidden size of 200 neurons. The training process was based on cross-entropy loss function, mini-batches, and Adam optimizer. The dropout of the softmax layer is set to 0.2 to avoid over-fitting. To evaluate the model’s performance, we shuffle the data and split the dataset into a training set, a validation set, and a test set, containing 80%, 10%, and 10% of the trajectories, respectively.

To evaluate the results, we compared the path prediction accuracy with vanilla RNN, CBP, and GatedCNN. These methodologies represent traditional methods in time sequence processing, old works in SRS, and the convolutional approach.

**Vanilla RNN**. The predominant approach to time sequence processing is Hidden Markov Model (HMM). The Baum–Welch algorithm, the standard algorithm for HMM, does not work well in path prediction tasks [28]. We use vanilla RNN instead of HMM. These two methods are similar in the mathematical structure and describe the dependency between time sequence through the hidden state. To imitate HMM in [28], we construct a $\left(n_{cells},2\right)$ embedding matrix from the geographic information system directly. The first dimension corresponds to the number of cells. The second dimension contains the latitude and longitude of the cell centroid. The geographic coordinates are converted to the Universal Transverse Mercator coordinate system. Specially, we use WGS 84/UTM zone 52N (EPSG: 32652) to compute and visualize the data in this work. The vanilla RNN uses spatial embedding, a block of one layer having a hidden size of 200 neurons, and a softmax decoder which is the same as the LSTM model.

**Collective Behaviour Pattern (CBP)** [8]. The CBP is a real-time and incremental mining method [8] that can be used for grid-based path prediction. Spatial-temporal points associated with the user’s behavior are mapped to the nearest gird cells and put into a greedy algorithm to determine heuristic solutions using empirical knowledge. The CBP is a simplified version of the discrete-time Markov chain. The main difference lies in the probability matrix of CBP and is updated with one order time sequence and limited by spatial relationship.

**Gated Convolutional Neural Network (GatedCNN)** [49]. Gated mechanisms control what information should be propagated through the hierarchy of convolutional layers. Stacked convolutions can extract hierarchical features over large contexts to increase effective temporal representation in parallel [50]. We use a kernel size of five for all the kernels. One gated convolutional layer is stacked after the word2vec embedding layer. After this, there are another 15 gated convolutional layers in 3 residual blocks. The final classifier consists of a dropout layer, a fully connected layer, and a softmax layer.

### 4.3. Evaluation of Geo-Embeddings

**Figure 6:**
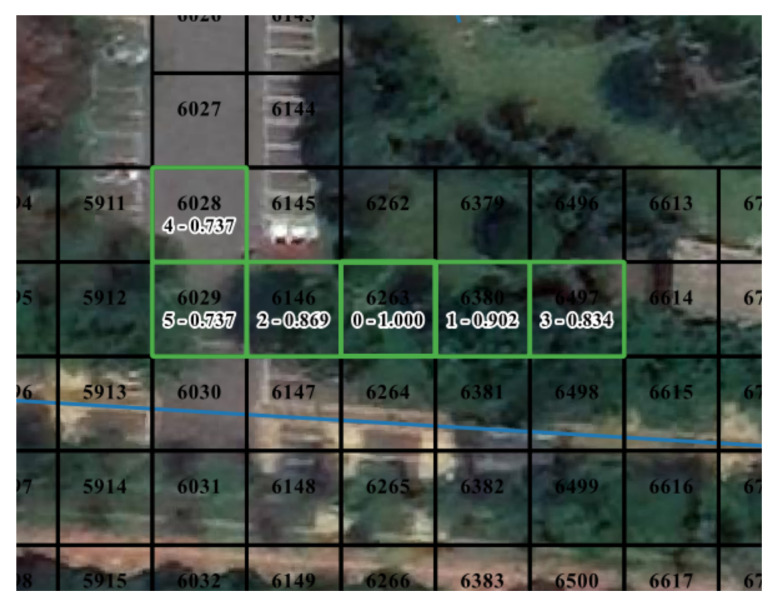
Example of top five proximal cells of CELL6263. “0-1.000” indicates that it is the target cell and the cosine similarity to itself is 1.000. “1-0.902” denotes that CELL6380 ranks 1 in proximal cells, and the cosine similarity to the target cell is 0.902.

Geographical distance and behavioral distance are not proportional: the same distances in space do not imply the same behavioral similarities. Although cells’ intervals are geographically comparable, human’s motion between them may be different. It means a feasible approach to mitigating the problem of the curse of dimensionality. That is, replace the original cell index with meaningful high-dimensional vectors. Figure 6 reports the geographic coordinates of cells and the top five proximal cells of CELL6263. The rectangular area formed by top-left CELL5913 and bottom-right CELL6616 is a vehicular road. There is a sidewalk under the tree from CELL5912 to CELL6614. For a pedestrian at CELL6263, the possible movement pattern is walking along the sidewalk or turning to CELL6028 to enter the car park. Their behavioral representation is different. It implies that the behavioral relation changes. The dense vector received by word2vec can represent the behavioral characteristics of the cell.

**Figure 7:**
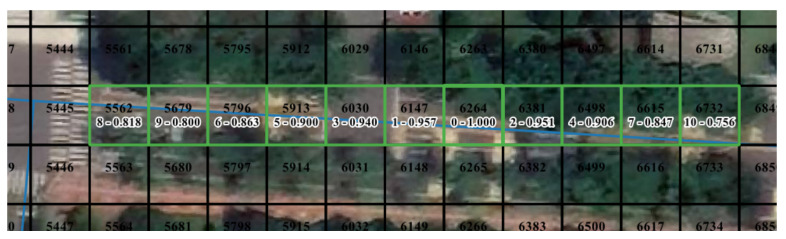
Example of top ten proximal cells of CELL6264. The cell is under the open sky. “0-1.000” indicates that it is the target cell and the cosine similarity to itself is 1.000. “1-0.957” denotes that CELL6147 ranks 1 in proximal cells and the cosine similarity to the target cell of 0.957.

The movement pattern of pedestrians is complicated. Users can work in various spaces on campus, such as roads, grasslands, squares, and buildings. By comparison, driving is restricted by the road and has more obvious regularity. Meanwhile, GPS-enabled mobile devices are typically accurate under the open sky. It allows spatial-temporal data to express movement behavior accurately. Cells on roads can reflect the difference between behavioral distance and geographical distance. Figure 7 shows the top ten proximal cells of CELL6264. Cells on the road tend to have very high similarity values with cells along the road and low similarities with both sides of the road. In other words, cells belonging to the road are maximally connected to nearby locations along the road.

**Figure 8:**
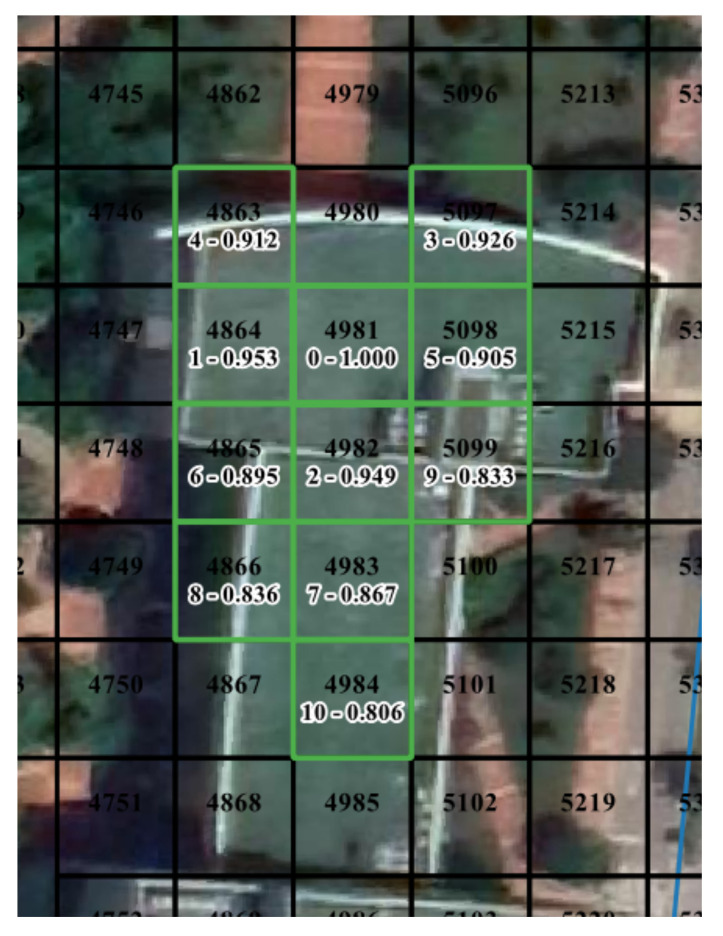
Example of top ten proximal cells of CELL4981. The cells are indoors. “0-1.000” indicates that it is the target cell and the cosine similarity to itself is 1.000. “1-0.953” denotes that CELL4864 ranks 1 in proximal cells and the cosine similarity to the target cell of 0.953.

In contrast to that, pedestrians tend to perform frequent short-distance movements in buildings, such as from one room to another. Furthermore, GPS-enabled mobile devices’ accuracy worsens indoors. These two reasons make it difficult to learn behavior patterns from many irregular movements or erroneous position data in buildings. As depicted in Figure 8, the cells indoors have high similarity values with cells in the surrounding area. For CELL4981, the behavior distance and the spatial distance are similar.

**Figure 9:**
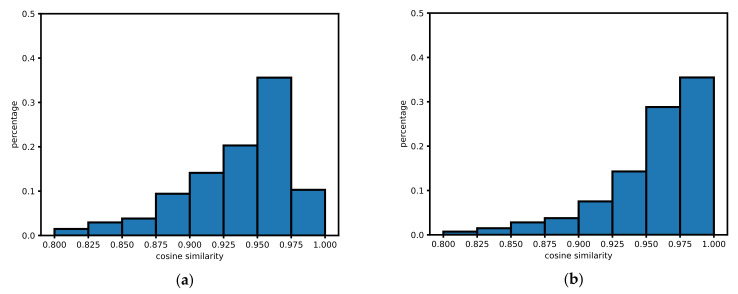
Similarity distribution of (**a**) buildings and (**b**) roads.

Considering the movement restrictions and the GPS accuracy on the road and in the building are the two extreme cases of studying the movement patterns. The road restricts the movement, and the GPS accuracy is high. On the contrary, the activities in the building are random, and there are a lot of erroneous data received from GPS. We compare the spatial distribution of the top ten similarities of the cells in the two cases. Roads and buildings are obtained from OpenStreetMap. For each cell in the research area, if the cell intersects a multiline string geometry of the road, it is considered to be on the road. Similarly, suppose a cell intersects with a multipolygon geometry of a building, it is considered to be inside the building. The overall different similarity behavior of buildings and roads can be observed in the normalized histograms of Figure 9. Both of the two cases tend to have significant percentages of high similarities. For a specific cell, the location of the cell cannot be distinguished by the distribution of the top ten similarities.

**Figure 10:**
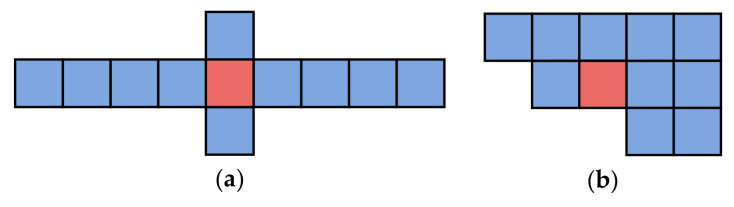
Example of two positional relationships when the average distances are (**a**) 22.0 m and (**b**) 16.0 m. The average distance is the average of the distances from the center of the target cell (red) to the centers of other cells (blue).

We measure the average distance of cells with their top ten proximal cells. The results are reported in Table 1. The difference is noticeable. Cells on the road have a higher sparsity. Figure 10a shows an example of a cell’s positional relationship with the top ten proximal cells. The average distance, in this case, is 22.0 m. Cells with similar behavioral distances tend to extend in specific directions. The behavior distance is a noticeable difference from the geographical distance. Figure 10b is an example that the average distance is 16.0 m, close to the average distance of the cells in the building. In this case, the cells are clustered together, and the positional relationship is close to the geographic distance. The behavior distance is identical to the geographical distance approximately. It means the behavior pattern tends to be random. The word2vec model is an effective embedding method in behavior representation.

### 4.4. Evaluation of Path Prediction

We evaluate the LSTM model with three other typical path prediction methods. Table 2 reports the comparison results in terms of macro-recall and weighted-recall. The recall is the fraction of true positives out of the actual positives. It measures the ability of the model to find all the positive units in the dataset [51]. Macro-recall is simply computed as the arithmetic mean of the metrics for single cells. Weighted-recall is the recall that has been weighted by the frequency of each cell. LSTM outperforms the other methods. Compared to CBP in previous work, the two recalls are improved by 0.34 and 0.23, respectively. For vanilla RNN, macro-recall and weighted-recall improve by 0.50 and 0.38. GatedCNN has the most significant number of parameters among the four methods but does not achieve well. Its weighted recall is 0.15 lower than LSTM.

The vanilla RNN here is only to imitate the mathematical structure and results of HMM and has not undergone the necessary hyperparameter tuning process in a modern neural network. It reports significantly lower accuracy than others. The results are close to Wesley Mathew [28], who used the Baum–Welch approach to estimate HMM parameters. It suggests that traditional HMM without manual feature extraction for time sequence multi-classification can not capture the complex sequential transition from historical trajectories. CBP is the baseline for path-based prediction of SRS. It has a discrepancy between macro-recall and weighted-recall, which indicates that the frequency of occurrence of different cells within the dataset is different. Cells in areas of participants’ daily movements frequently appear; on the contrary, in regions that participants cross occasionally appear rarely. The training data has more frequently appearing cells as the results lead to more accuracy. The differences in prediction accuracy for different regions of cells, multiplied by the weighting factor, lead to differences between macro-recall and weighted-recall. The weighted-recall of GatedCNN is higher than CBP, and the macro-recall is lower. It implies that CBP and GatedCNN capture different behavior patterns. CBP is based on the first-order Markov model and only considers the effect of the current cell on the predicted outcome. However, GatedCNN extracts a whole sequence through stacked one-dimensional convolutional layers, overfits more than CBP and LSTM on a small dataset, and improves the overall accuracy but loses the ability to generalize various behavior patterns.

Moreover, we analyze how different trajectory characteristics affect the prediction. Figure 10 and Table 1 show that the positional relationship between a cell and its top ten proximal cells can represent the user’s behavior pattern. It can be measured by the average distance from the cell’s centroid to the centroid of similar cells. Therefore, we use the average distance to classify cells in the research area and study the influence on the prediction. Table 3 compares the experimental results on weighted-recall and macro-recall. For the case where the average distance is less than 15 m, the behavior pattern of the cell mainly reflects randomness. The four methods all obtain the lowest accuracy. It is a challenge to predict random movement. As the average distance increases, the prediction accuracy increases, reaching a crest when the average distance is between 25 m and 30 m. At this time, the positional relationship of cells is close to Figure 7. Users move along a fixed route with regularity. When the average distance is greater than 30 m, prediction accuracy is slightly reduced. As shown in Figure 7, there may be two situations where the average distance is greater than 30 m. The first case is that, based on Figure 7, the cell is not located in the center of the top ten proximal cells. It is caused by the imbalance in the direction of movement of users passing through this cell. Considering that our dataset has been collected for a long time, the number of occurrences of this imbalance is small. Another case is that the speed of users is high, causing the distribution of the top ten proximal cells to be discontinuous in geographical space. The time interval at which we collect spatial-temporal data is 5 s. The side length of the cell is 10 m. If the user’s moving speed is greater than 7.2 km/h (2.0 m/s), the cell sequence mapped may be discontinuous. For the cell on the motor traffic lane, the user’s speed is usually much greater than 7.2 km/h. The trajectories passing through the cell are erratic, resulting in a discontinuous distribution of the top ten proximal cells. The discontinuous trajectories increase the selectable range of the prediction, resulting in a decrease in prediction accuracy. Together with the four methods, LSTM has apparent advantages both in terms of weighted-recall and macro-recall.

Intuitively, traveling through the same distance (in this work, 18 cells), if the displacement is large, it means that the user is moving in a particular direction. The next position should be easy to predict. Conversely, if the displacement is small, the user is repeatedly moving in an area. It is difficult to predict the user’s next position. Table 4 presents an overview of weighted-recall and macro-recall comparisons for different values of traveled displacement. When the displacement is less than 50 m, the trajectory is restricted to a small area, the weighted-recall and macro-recall of LSTM are close. The results indicate that the feature extraction ability of LSTM is stronger than the other three methods. As the displacement increases, the accuracy continues to improve, reaching a peak of 0.7165 when the displacement is between 150 m and 200 m. The most effortless trajectory to predict is to go straight. The displacement corresponding to 18 cells arranged in a straight line is 170 m, located in 150 m to 200 m. It is consistent with intuition. The displacement continues to grow. When the displacement is greater than 200 m, it indicates that the user’s moving speed is fast and that the trajectory’s cells are not continuous. As mentioned in the previous paragraph, a too-fast speed will increase the selection range of the predictions and reduce the prediction accuracy. Comparing the four methods, LSTM is in a leading position in both weighted-recall and macro-recall.

### 4.5. Evaluation of Service Provide Rate

To evaluate the actual performance of different path prediction algorithms in SRS, we consider the service provision rate for sensor counts of 20, 50, 100, 200, and 300, respectively. Table 5 displays the basic statistics for sensor network communication disks. The number of sensors directly affects the sensor communication disk coverage rate, which is 0.052 for 20 sensors and increases to 0.560 for 300 sensors. The number of sensors is essentially a scaled parameter to adjust the coverage. It is then used to test the performance of the SRS under different coverage scenarios.

Table 6 presents the service prevision rate comparison for a different number of sensors. $R_{G}$ is the GPS reliability. The ideal scenario of a completely accurate mobile device position determines the upper limits of the service provision rate, and the lower limit is the case when GPS positioning is sometimes wrong ($R_{G}=0.9$). The service provision rate reinforced by dual collaboration strategy is distributed between the ideal scenario and the actual scenario.

A cell occupies a square space in the research area. Rarely, a sensor network can completely cover a specific cell. Therefore, even with the metadata provided by SRS containing the correct correspondence between the cell and the sensors, there is no guarantee that a successful connection can be made within the cell. The service provision rate for the ideal case is not 1.0.

The four models are characterized separately. Our model (LSTM) has the best performance. By applying the dual collaboration strategy with LSTM, the effect of mobile device position error can be compensatory. Unlike the version of recall, Monte Carlo simulations show that the second well-performing method is CBP. The predicted output of CBP is chosen from the eight cells surrounding the input cell. Given the daily movement speed of participants, the true cell is generally near the input cell. Even if the predicted cell is not the same as the true cell, it is still near the true cell in geographical space. A sensor may cover both true and predicted cells. In the evaluation of service provision rate, the possibility improves the performance of CBP.

The vanilla RNN behaves quite differently from weighted-recall. As shown in Table 6, it performs slightly better than GatedCNN, both of which are effective time series multi-classification models. However, the features extracted are different. GatedCNN focuses on patterns of high-frequency sequences and improves weighted-recall by enhancing the accuracy of high-frequency sequences. The vanilla RNN extracts directions [52] from sequences and cannot precisely predict cells. However, the predicted positions are close to the true cells. A sensor covers not just a single cell but several cells within a circle. With the predicted and true positions being spatially adjacent, there is a chance that the corresponding two cells are in the same sensor communication disk. Imperfect predictions can provide a successful service. Therefore, as with CBP, vanilla RNN is significantly superior to that of the weighted-recall in Table 1. Both vanilla RNN and GatedCNN improve the service provision rate of dual collaboration strategy, with the former being slightly more effective.

**Figure 11:**
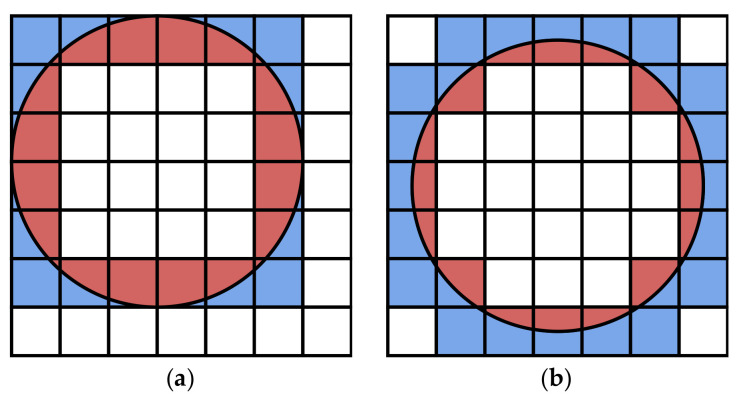
Two examples of the positional relationship of the cells and the communication disk. The colored cells intersect with the border of the communication disk. When a user moves in the colored cells, the red area is proportional to the service provision rate. The ratio of the red area to the colored cells is (**a**) 0.613 and (**b**) 0.303.

The relation between the number of sensors and the service provision rate can be used to measure the reliability of the simulation results. When m=0, the users in the cell do not need SRS service. In the simulation, we ignore these cells, as shown in Equation (8). Theoretically, the communication disk coverage rate or the number of sensors should not affect the final service provision rate. However, Table 6 shows a difference in the service provision rate for the different number of sensors. The difference comes from the stochastic behavior of the Monte Carlo simulation. As shown in Figure 4, the sensors and their corresponding communication disks are randomly generated at each step in the simulation flow. Their positions affect the connectivity in the cells at the edge of the communication disks. Figure 11 shows two examples of positional relationships between cells and communication disks. In Figure 11a, for the colored cells, using the area ratio to calculate the service provision rate in these cells obtains 0.613. Correspondingly, the case of Figure 11b corresponds to the value 0.303. Each step is performed using the same historical trajectories but with different sensors in different positions, which leads to fluctuations in the results. In Table 6, the differences between the maximum and minimum values of service provision rate for the six scenarios are 0.010, 0.013, 0.011, 0.014, 0.011, and 0.010, respectively. Taking the service provision rate at 0.670 as a reference value, the deviation of 0.014 is only 2%, and Table 6 can be considered as a qualified result. If the number of simulations $N_{s}$ is further increased, the results may be more consistent, but it will waste computational resources. Therefore, we set $N_{s}=100$, and Table 6 is used for the service provision rate of the different scenarios.

### 4.6. Discussion

The word2vec algorithm is a popular method for building dense vectors and is widely used in natural language processing. We apply it to analyze human behavioral patterns. Based on human movement behavior, the spatial relationship between cells is ignored. Only the movement from one cell to another cell is taken to construct the embedding vector. After evaluation, the behavioral and spatial distances between cells are disproportionate. The finding is similar to the existing work called geo-embedding [33]. Some cells may be spatially adjacent but behaviorally distant. For example, the behavioral distance between the cells along the road is proximity. The behavioral distance between the road and the space on both sides is distant. It indicates that the vector, after embedding, can represent human behavior patterns. The representation is based on the frequency of other cells appearing around the target cell. It is the same as the word2vec model. By comparing the positional relationship of the top ten proximal cells, we notice that the positional relationship in embedding space can represent human behavior. Suppose a person frequently follows a specific trajectory; the shape of the top ten proximal cells’ position is similar to the trajectory, as shown in Figure 6 and Figure 7. Conversely, there is no particular direction when the cell is in a square or indoors where GPS noise is severe. The spatial and behavioral distances between this cell and nearby cells are proportional. The top ten proximal cells tend to group, as shown in Figure 8. We extract the building layer and the road layer from OpenStreetMap. By checking whether the cells intersect with the multiline string geometry from the building or road layers, the cells are categorized into building cells, road cells, and other cells. We calculate the average distance between a target cell and its top ten proximal cells to obtain Table 1 and then draw two typical positional relationships, as shown in Figure 10. Table 3 shows the recall comparison for different values of average distance. The columns where the crests and troughs are found confirm the guesses in Figure 10. The results imply that the average distance to the top ten proximal cells can indicate humans’ behavior patterns. When the average distance is small, human’s behavior reflects randomness. Relatively, when the average distance is larger, the behavior shows regularity.

The dense vectors obtained after embedding are used as inputs for the neural network for path prediction. We compare the accuracy of four path prediction algorithms, which are vanilla RNN, CBP, GatedCNN, and LSTM. The Hidden Markov Model was the most popular algorithm for handling time series in the past. For path prediction, it requires the manual design of hidden states. Learning with the Baum–Welch approach does not provide meaningful results. We replace the HMM with a vanilla RNN without hyperparameter tuning as the baseline for path prediction. CBP is the method applied in the existing SRS. GatedCNN supports increased parallelism and effective temporal representation and is a promising topic. The LSTM has been employed in several works and is the most widely used deep learning-based path prediction algorithm. We compare macro-recall and weighted-recall. The discrepancy between macro-recall and weighted-recall indicates an imbalance in the distribution of our dataset. Furthermore, it shows the differences in path prediction algorithms. The GatedCNN is similar in accuracy to work [50]. Still, the learned features are different from the RNN-based approach. Analogous to the higher-order Markov model, it captures the regular historical trajectories from high frequencies, with macro-recall and weighted-recall differing the most. With the comparison of recall, we realize that LSTM is a suitable algorithm for path prediction in SRS.

We also compare the performance of historical trajectories with different complexities under various path prediction algorithms. To characterize the complexity of the trajectories, we tried to use features from two spaces. In the embedding space, the average distance to the top ten proximal cells is used. In geographical space, the displacement from the starting cell to the end cell is also considered a feature. The results show that too long or too short a displacement from the starting cell to the end cell reduces the prediction accuracy. In contrast, the average distance to the top ten proximal cells is proportional to the predictability. Therefore, the average distance is a helpful feature to evaluate predictability. The finding is beneficial for studying human behavior patterns, for example, visualizing human behaviors at different cells in a geographic information system.

Path prediction is introduced to improve the performance of SRS. High-accuracy path prediction algorithms do not always perform well in the real scenario. We use the service provision rate to evaluate the performance of sensor filtering in SRS. In this work, we employ a dual collaboration strategy to improve the service provision rate. The position obtained by the mobile device from GPS is sometimes wrong. Furthermore, the second position, predicted by historical trajectory, may also be mistaken. Simultaneous attempts are made to connect the sensors corresponding to these two positions. If one of the positions is correct, the mobile device can connect to the actual ambient sensors and acquire heterogeneous data. However, there is a limitation. Its upper limit is the ideal scenario where the GPS is completely accurate. In comparison with four path prediction algorithms, the LSTM model almost reaches the upper limit, with a difference of only 0.003, achieving the purpose of compensating for the loss caused by GPS unreliability. It raises the question of whether it is necessary to improve the accuracy of path prediction by the attention mechanism [43,44]. In SRS, the improvement is estimated to be only 0.001. However, in other intelligent services, the increase may be meaningful. Meanwhile, the difference between the recall and the results of the Monte Carlo method illustrates the reasonableness of the Monte Carlo method for evaluating the performance of sensor filtering in SRS.

## 5. Conclusions

We addressed the problem of sensor filtering with incorrect GSP positions and quantitatively showed that the dual collaboration strategy was able to solve the sensor filtering problem. With the dual collaboration strategy, the sensor filtering module collaborates both positions from GPS and path prediction. If one of the positions is correct, the mobile device can connect to the actual ambient sensors and acquire heterogeneous data. The path prediction module consisted of geo-embedding and LSTM to predict the current position of the mobile device from a historical trajectory. We also proposed a Monte Carlo-based simulation flow for evaluating the service provision rate. Several hundred thousand trajectories were collected. The experiment showed many new results compared to previous works: 1. The geo-embedding was also suitable for grid-based partition for user behavior feature extraction. In embedding space, the positional relationship was able to represent human behavior patterns. The average distance was used to recognize the predictability of the trajectories. 2. The path prediction algorithms with various structures focused on different characteristics of historical data. LSTM was a suitable model for grid-based path prediction. 3. We used the service provision rate to evaluate the performance of sensor filtering in SRS and employed the Monte Carlo-based simulation flow for quantitatively evaluating the service provision rate. Four path prediction models were evaluated under several scenarios for the service provision rate. For our dataset, LSTM and CBP had the best performance for sensor filtering in SRS. The LSTM-based path prediction was able to compensate for the loss of the GPS drift and was an effective sensor filtering model.

There are several future directions for our work. First, we currently only predicted the current position using spatial-temporal sequences. In the future, we will plan to embed multi-modal information with historical trajectories, such as time, semantics, and context to improve the prediction accuracy further. Second, we still want to learn human behavior patterns with specially designed neural networks to achieve better path prediction results. Third, we directly handled a fixed GPS reliability and communication radius to simulate the service provision rate in this paper. Building a more realistic model for better SRS evaluation is also an exciting topic. Finally, we will try to create a long-range path prediction model by the seq2seq approach to improve the sensor filtering effectiveness in SRS further.

## Figures and Tables

**Table 1:** Average distance with top ten proximal cells.

Buildings top 10 average distance	15.9 m
Roads top 10 average distance	22.8 m

**Table 2:** Overall performance comparison between our methodology (LSTM) and vanilla RNN, CBP, and GatedCNN.

	Marco-Recall	Weighted-Recall
Vanilla RNN	0.1037	0.2956
CBP	0.2620	0.4427
GatedCNN	0.1856	0.5296
LSTM	0.6057	0.6780

**Table 3:** Weighted-recall (and macro-recall in brackets) comparison for different values of average distance.

Average Distance=	≤15 m	15–20 m	20–25 m	25–30 m	>30 m
Vanilla RNN	0.1685 (0.0567)	0.2371 (0.0911)	0.4144 (0.0757)	0.4798 (0.0622)	0.2800 (0.0506)
CBP	0.376 (0.1512)	0.4183 (0.2181)	0.5025 (0.1576)	0.5465 (0.1243)	0.3721 (0.1103)
GatedCNN	0.4012 (0.0875)	0.5015 (0.1494)	0.6275 (0.0755)	0.6612 (0.0572)	0.4256 (0.0326)
LSTM	0.5754 (0.3098)	0.6646 (0.4866)	0.7333 (0.4424)	0.7563 (0.4042)	0.6738 (0.4178)

**Table 4:** Weighted-recall (and macro-recall in brackets) comparison for different values of displacement.

Displacement=	≤50 m	50–100 m	100–150 m	150–200	>200 m
Vanilla RNN	0.1039 (0.0747)	0.1362 (0.0816)	0.2695 (0.1042)	0.3729 (0.1124)	0.1052 (0.0534)
CBP	0.2016 (0.1745)	0.3017 (0.2036)	0.4340 (0.2813)	0.5015 (0.2813)	0.1825 (0.1340)
GatedCNN	0.1926 (0.1197)	0.3287 (0.1369)	0.5287 (0.1904)	0.6122 (0.2028)	0.1514 (0.0682)
LSTM	0.4546 (0.4456)	0.5797 (0.5393)	0.6859 (0.6193)	0.7165 (0.6031)	0.4528 (0.4030)

**Table 5:** Basic statistics of sensor communication disks.

Number of Sensors	20	50	100	200	300
Sensor communication disks coverage rate	0.052	0.126	0.236	0.419	0.560

**Table 6:** Service provision rate comparison for a different number of sensors.

Number of Sensors	20	50	100	200	300
Actual scenario ($R_{G}=0.9$)	0.606	0.607	0.613	0.612	0.616
Vanilla RNN	0.661	0.662	0.671	0.669	0.674
CBP	0.667	0.667	0.675	0.673	0.678
GatedCNN	0.658	0.660	0.669	0.667	0.672
LSTM	0.670	0.671	0.678	0.677	0.681
Ideal scenario	0.674	0.674	0.681	0.680	0.684

## Data Availability

Not applicable.

## Abbreviations

The following abbreviations are used in this manuscript:

IoT	Internet of Things
SRS	Sensor Registry System
HMM	Hidden Markov Model
RNN	Recurrent Neural Network
RSRP	Reference Signal Received Power
